# The participation of Wajãpi women from the State of Amapá (Brazil) in the traditional use of medicinal plants – a case study

**DOI:** 10.1186/1746-4269-8-48

**Published:** 2012-12-19

**Authors:** Nely Dayse Santos da Mata, Rosinaldo Silva de Sousa, Fábio F Perazzo, José Carlos Tavares Carvalho

**Affiliations:** 1 Curso de Enfermagem, Centro de Ciências Biológicas e da Saúde, Universidade Federal do Amapá, Rod. Juscelino Kubshketch, km 02, CEP 68902-280, Macapá, Amapá, Brasil; 2 Laboratório de Estudos Sociais, Universidade Federal do Amapá, Rod. Juscelino Kubshketch, km 02, CEP 68902-280, Macapá, Amapá, Brasil; 3 Setor de Ciências Farmacêuticas, DCET, Departamento de Ciencias Exatas e da Terra, Universidade federal de São Paulo – Unifesp, Rua Prof. Artur Riedel, 275, CEP 09972-270, Diadema, Sao Paulo, Brasil; 4 Centro de Ciências Biológicas e da Saúde, Laboratório de Pesquisa em Fármaco, Universidade Federal do Amapá, Rod. Juscelino Kubshketch, km 02, Macapá, Amapá, CEP 68902-280, Brasil

**Keywords:** Wajãpi women, Medicinal plant, Traditional knowledge, Sustainability

## Abstract

**Background:**

The purpose of this study was to analyze the importance of traditional medicinal plants use to Wajãpi women in the State of Amapá, Brazil, as well as their practices in the local common illnesses of treatment considering the prevailing practice by non-Indians.

**Methods:**

This study was conducted in the Community of the Wajãpi Indigenous People, a Brazilian territory located in the central western State of Amapá. Wajãpi women were selected for the interview since they have the responsibility to harvest, collect and prepare the preparations. The studied women were residents of four villages. The number of women within these four villages is 24.

**Results and conclusions:**

The findings fell into the following three categories: 1) The daily use of medicinal plants by women and main methods of application. In this category, the botanical families found included Leguminosae-Caesalpinoideae, Anacardiaceae, Meliaceae, and Rubiaceae. The main forms of use found were teas, baths, maceration, *in natura*, and juices; 2) Through analysis of illness and treatment records, a lack of knowledge integration in the health system was shown to be due to a variety of gaps and the need of health professionals to be more aware about the local culture which they intend to work with, what could decrease the prevailing barriers between the social groups involved; 3) Traditional knowledge and possible sustainability can be fostered by stimulating the transmission of traditional knowledge from generation to generation, therefore reducing the dependence on industrialized medicines and also by maintaining an appreciation of those practices among youngsters, who tend to question them.

## Background

Brazil is a promising environment for studies with medicinal plants because of its huge and different areas (Rain Forest, Savannas), and its broad cultural and biological diversity. Brazil is inhabited by rural and urban populations of 232 indigenous ethnic groups, 1,342 Quilombola groups (descendants of Afro-Brazilian people), and mestizo groups derived from the miscegenation of Indian, Africans, European and Asian people [1].

The indigenous society’s integration with the surrounding society has generally occurred through large projects and infrastructure development. These policies have been harmful to indigenous groups, either through the degradation of their secular way of life and culture, or by putting their survival in jeopardy. Considering they live with different healthcare forms, they have created a sort of interaction between these forms although indigenous youngsters tend to use healthcare forms that are more characteristic of non-Indians due to governmental support. This causes the abandon or lack of interest in traditional medicine forms provided by Wajãpi women. Nonetheless, the community as a whole has maintained centuries-old traditional habits, like treating illnesses with medicinal plants preparations.

The Wajãpi Indians are originally from the lower Xingu region. In the beginning of 18th century they had begun to migrate to Northeastern Amapá (Brazil) and Southern French Guiana, region where they live nowadays. The Wajãpi is a traditional community which speaks Tupi-Guarani language and is distributed around the geographical limits of Brazil and French Guiana borders. Today, the population of this indigenous group is 847 people [2].

The first contact with the Wajãpi tribe was made in the beginning of the last century with the Amazonian riverbank population. Contact was made by donating objects such as pans and agricultural tools [3], aiming to increase the agricultural production and decrease possible diseases.

In this way, the Wajãpi’s consider that illnesses are provoked by a spirit that punishes people who have violated one of many prohibitions related to food, sex, and above all, hunting. In the case of hunters, the maleficent influence of the spirits is primarily towards the children.

Other indigenous groups have made research advances with medicinal plants during the course of their traditional use. One of these groups is the Kraho Indian tribe, which is located in the Kraolândia reserve, in the State of Tocantins, Brazil. Studies with medicinal plants are also advancing in other groups. It is important to highlight the participation of indigenous women, who are partly responsible for passing this wisdom, which have been developed for millennia, to their descendants.

This study emphasizes practices using the traditional knowledge of Wajãpi women regarding medicinal plants.

## Methods

### Research site

This study was conducted in the Community of the Wajãpi Indigenous People, a Brazilian territory located in the central western State of Amapá, in the counties of Laranjal do Jarí and Pedra Branca of Ampari, Brazil.

### Ethical research considerations

The research project was submitted with prior consent of the Wajãpi indigenous community General Assembly, Funai Regional and FUNAI/Brasilia, CONEP and CGEN/NMA. Approval was given under the grant number 244/CGEN/MMA.

All requirements set by the Provisionary Measure nº 2.186-16 from August 23, 2001 and following Resolution nº 05 of the Council of the Genetic Patrimony Management – CGEN, Brazil, were meet.

All participants were informed that data was being collected for research. To guarantee the physical and emotional well being of these women, the used research information was treated in a confidential manner. They were identified using subjects, the letter “M”, followed by a number in ascending order “M_1,_ M_2_ …”, thereby ensuring personal privacy.

Participants were allowed to dropout during any phase of the study, and access to the research results was guaranteed. The interviews were recorded after informed consent was obtained from the volunteers.

### Research subjects

Wajãpi women who prepare medicines using medicinal plants and administer them to the community were selected. The female population of the entire indigenous area, 424 inhabitants, is distributed among 42 villages. Wajãpi women were selected for the interview since they have the responsibility to harvest, collect and prepare the preparations. Wajãpi men have responsibility related to hunting and defending the village.

**Figure 1:**
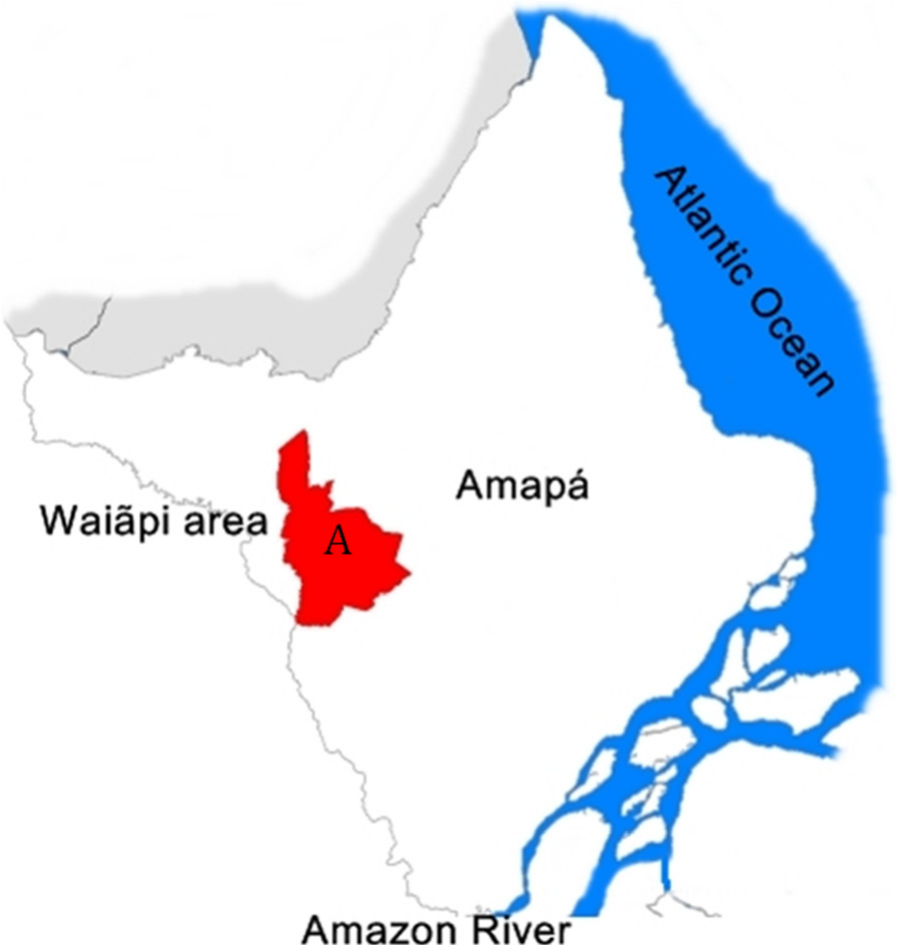
Region habited by Wajãpi indigenous community marked with the letter A.

The studied women were residents of four villages: the triangle community of Amapari-CTA; Manilha; Cachoeirinha, and Jakaré (Figure 1, letter A). The number of women within these four villages is 24. The youngest women respecting the experienced advice of the elder women had declined to participate. Among the elder women, 13 decided to participate in the research, 5 dropped out during the study, with eight respondents all over the research.

### Obtaining ethnographic data

Semi-structured interviews were obtained for personal data (name, sex, age, religion, marital status, place of birth, school grade) as well as ethnopharmacological issues (name of natural resource, use, part used preparation, administration route, side-effects, doses, and restrictions).

Field notes were used for data collection. The cultural scene and reflections about the importance of traditional practices regarding the treatment of community members by Wajãpi women with medicinal plants were described. These records were based on observations regarding the use, rituals, and particular expressions of the participants related to the study. This technique was used throughout the research process.

The determination of the women’s conception of the research objective involved a semi-structured interview, scheduled for each woman who agreed to participate.

Interviews were recorded, offering a richer content for the research. These interviews took place at the village where they lived during the time of study. The subjects (elder women) were accompanied with a female indigenous interpreter who takes part in their daily life. The younger indigenous women understand and speak Portuguese.

### Collection of botanical material

Botanical collection was conducted after the women had been interviewed. The information and the main medicinal plants used for treating sick people were grouped together. It had been collected 19 samples for the confection of exsiccates. Samples were sent to the herbarium of the “Instituto de Estudos e Pesquisas do Estado do Amapá - IEPA (HAMAB)” for botanical identification.

Medicinal plants that are located near their domiciles are usually planted and collected by women. For other plants mentioned that were not found in surrounding areas, the women recommended that the indigenous man Matã Wajãpi accompanied them into the forest for identification. According to the women, Matã was also known as “Dr. Root”, given his vast knowledge of the area.

The techniques used for collection of the botanical material followed the descriptions by Fidalgo and Bononi [4]. Taxonomic classification was conducted using the Tropics Database of the Missouri Botanical Garden. The identification and classification of botanical material was conducted by technicians and specialists from the “Divisão de Botânica do Instituto de Pesquisas Científicas e Tecnológicas do Amapá – IEPA”. The specimens were classified according to Cronquist [5].

### Data analysis

Eight indigenous women conveyed their narratives and seven participated as oral translators when necessary. The names of the plants were reviewed several times with regard to their spelling in the Wajãpi language by the women who knew how to write and who understood Portuguese. Later, another review was completed by an indigenous professor, Caubi Wajãpi, to confirm the indigenous spelling.

During analysis of the material obtained, three categories were highlighted: 1) Medicinal plants used daily by the women and their main methods of preparation and use; 2) Narratives regarding illness and treatment (Have indigenous and non-indigenous knowledge been integrated considering the use of medicinal plants or synthetic medicines from the govern?); and 3) Traditional knowledge and possible sustainability.

Data regarding the frequency of occurrence was described in absolute (number of times mentioned) and relative (percentage) terms.

## Results and discussion

### Social characterization of indigenous women and research participants

The participants were married women ranging from 31 to 74 years old. The number of children varied from 1 to 6, and no gender predominated among the children of these women. The majority of the respondents had not attended school although all the women who served as interpreters had attended school.

Their daily activities consisted of looking for food, working in the fields, hunting with their respective spouses, taking care of domestic animals, searching for natural supplies to make crafts, and participating in food and drink preparation for parties and traditional rituals.

### Medicinal plants used daily by the women and their main methods of preparation and use

Medicinal plants are used by almost all adult women. Nonetheless, knowledge is diffused throughout the community. This was evidenced by the acquired experience of the older women. When asked about their knowledge of medicinal plants, the younger women always affirmed that they did not know much and that only the older women were experts on the subject. The main plant family used by the Wajãpi in this study was Leguminosae, with five plants identified.

The use of plants by Wajãpi women is practiced mostly among the women’s own families, and there is no need for remuneration if other families need it. The women do not worry about storing the parts of plants used as medicine in their home because the majority of the plants mentioned are used fresh (*in natura*). The plants are easily found usually close to their home or village. Depending on the illness, other plants can be searched in the forest. However, if the plants grow far away, the women ask a man from the group who knows how to find it to collect it for them, or ask their husbands to accompany them because of animals in the forest.

A variety of herbs, roots, barks, and leaves have been used in the traditional medicine of indigenous women as well as different forms of preparation. Many of these medicines are prepared by maceration using alcohol or water, smoke inhalation, or water infusion for various types of diseases and illnesses.

For the Wajãpi, humans are not the “owners” of natural resources surrounding them. Each species group has an “owner”, a spirit or a forest protector, and any action taken upon these groups requires mediation and/or prudence in their use [6].

Considering plants as natural resources, they also have “owners”, which are not human. It can be a spirit of or even a bigger specie, like huge trees. Some trees are controlling masters. Some are more or less aggressive, and some, such as Kumaka (sumaúma – *Ceiba pentandra* L.) and Peyryry (angelin – *Dinizia excelsa*), must not be cut. If this tradition is violated, then the responsible for it will suffer consequences.

The way that plants are used has been passed from generation to generation by these women. This transmission is oral, as Wajãpi tradition dictates, and they are used cautiously. This helps to avoid reduction in the number of plants or even their complete disappearance, as when an unrelenting act of modernity occurs [7]. The following quote regarding plant use may be examined:

"“For urinary pain, cut “part” of the root of the taperebá and drink the water that comes from inside of it.” (M_3_)"

Several forms of preparation and use are reported. The most common include the use in baths or ingestion, maceration, or inhalation (Table 1).

**Table 1:** Botanical and ecological characteristics of the plants used as medicine by Wajãpi women (Amapari Indigenous Area-AP, Brazil).

**Indigenous Name**	**Scientific Name**	**Family**	**Characteristic**	**Voucher Number**	**Use**
Apukuapoã	*Psychotria colorata*	Rubiaceae	Shrub	AP2645	Earache
Jenipapo	*Genipa americana* L.	Rubiaceae	Arboreal	AP1984	Earache
Jiruru	*Parkia* sp.	Leguminosae-Mimosoideae	Arboreal	AP1254	Culinary
Janyro- Andiroba	*Carapa guianensis* Aubl.	Meliaceae	Arboreal	AP1425	Remove ticks and lice, general pain
Jamarata	*Zengibre officinalis* L.	Zingiberaceae	Herbaceous	AP2241	Infections
Mastruz	*Chenopodium* sp.	Chenopodiaceae	Herbaceous	AP3412	Abdominal pain
Peyryry	*Dinizia excelsa*	Leguminosae- Mimosoideae	Arboreal	AP1412	Abdominal pain
Perovyu or Perauvu	*Aspidosperma* sp*.*	Apocynaceae	Arboreal	AP0873	Warts topics
Kupa’y	*Copaifera* sp.	Leguminosae - Caesalpinoideae	Arboreal	AP0259	Infections, cicatrizing, insect bite
Kadjurá	*Bauhinia* sp.	Leguminosae- Caesalpinioideae	Shrub	AP0436	Bloody diarrhea, abdominal pain
Kuremó	*Chondrodendron* sp.	Menispermaceae	Liana	AP2657	Topic use for headaches and itching
Kaiju ou Akaju	*Anacardium* sp.	Anacardiaceae	Shrub	AP0679	Diarrhea, skin wounds
Kupa’i ou Kupuai’y	*Theobroma* sp.	Sterculiaceae	Shrub	AP1623	Culinary
Touriri	*Inga* sp.	Leguminosae-Mimosoideae	Arboreal	AP1835	Contraceptive
Tapereba Rapo	*Spondias mombin* L.	Anacardiaceae	Arboreal	AP1559	Urinary pain
Tatapotá	*Aniba* sp.	Lauraceae	Arboreal	AP1232	Diarrhea and abdominal pain
Urucu	*Bixa orellana* L.	Bixaceae	Shrub	AP0541	Insect repellent and bites
Yvyramemyry	*Miconia* sp.	Melastomaceae	Shrub	AP1147	Urinary pain
Yvyratai Wemy’a	*Sterculia* sp.	Sterculiaceae	Arboreal	AP1954	Abdominal pain

Thirty-seven plants were identified in Wajãpi indigenous therapy. Twenty-five of the most frequently mentioned plants are listed in Table 1. Fourteen samples were collected and forwarded to the IEPA/AP for taxonomic identification and deposite in the institution’s herbarium in the name of the Wajãpi community. They belong to approximately eleven families. Of these eleven, the scientific names of fourteen plants were identified. Therefore, the study documented twenty-one identifiable samples.

Table 1 shows the botanical characteristics and habitat of the main plants used for medicinal purposes by the women, who are classified as shrubs, arboreal, and herbaceous plants. It can be observed that the arboreal group of plants was predominant (10 citations), followed by shrubs (5 citations), and finally herbaceous plants (2 citations) (Table 2).

**Table 2:** Absolute and relative frequencies of medicinal plant families used by Wajãpi women (Amapari Indigenous Area–AP).

**Family**	**Absolute Frequency**	**Relative Frequency (%)**
Apocynaceae	01	2.40
Anacardiaceae	05	12.00
Bixaceae	02	4.76
Chenopodiaceae	03	7.14
Lauraceae	01	2.40
Leguminosae-Caesalpinioideae	10	23.8
Leguminosae-Mimosoideae	02	4.76
Meliaceae	05	12.00
Menispermaceae	04	9.52
Melastomaceae	01	2.40
Rubiaceae	05	12.00
Sterculiaceae	01	2.40
Zingiberaceae	02	4.76
Total	42	100 %

The plants mentioned by the Wajãpi women are represented by their respective families (Table 2). The indigenous women mentioned the following families: Leguminosae-Caesalpinoideae (Kadjurá and copaíba) (23.8%); Anacardiaceae (Kaiju), Meliaceae (Andiroba), and Rubiaceae (Apukuapoã and jenipapo) (11.9%); and Menispermaceae (Kuremó) (9.52%).

From the Leguminosae-Caesalpinoideae family, the Kadjurá and the Kopa’i plants are given the vernacular name of copaiba. It produces aqueous and clear oil. This oil, mixed with annatto dye (*Bixa orellana* L*.*), has been used by Wajãpi women for protection against insect bites or injuries. Referred to as “copaíva” or “copahu” by indigenous people (from the Tupi words: Kupa’iwa and Kupa’u, respectively), copaiba oil was used quite often among Brazilian Indians when the Portuguese arrived in Brazil [8].

Kadjura is an ascending shrub. In Brazil, this plant is widely distributed and is popularly called “monkey staircase” or “climbing staircase” [9]. Wajãpi women use this plant for bloody diarrhea and to decrease abdominal pain. Since both plants have been described as tannin rich species, the use of these plants can show healing and analgesic actions.

Cajueiro (Anacardiaceae) has been known by several names derived from the original Tupi language (acayu): acaju, acajaíba, acajuíba, caju-comum, cajueiro-comum, cajuil, caju-manso, cajuzeiro, and ocaju [10]. The Wajãpi women uses the bark from the trunk in the form of tea for diarrhea as well as for healing superficial skin wounds, triturating it and pressing straight to the injured area.

From the Meliaceae family, *Carapa guianensis* Aublet was noticed. This plant has been commonly known as “andiroba”, a vulgar designation derived from the indigenous words “nhandi” – oil and “rob” – butter [11]. Oil mixed with urucum dye (*Bixa orellana* L.) has been used by indigenous people as an insect repellent. The Wajãpi and Palikur use the oil to remove ticks and lice [12] and the Wajãpi women have reported using the oil for earaches, muscular and bone aches.

In the Rubiaceae family, apukuapoã and jenipapo have been described. According to the Wajãpi indigenous women, these plants are used for earaches. Genipapo is the fruit from the Genipapo tree. In Tupi-Guarani, genipapo means “fruit that is used to paint”. The Indians use the juice of the fruit to paint the body. The ink remains for several days and protects against insects.

In the indigenous area of the Amapari County, the women mentioned Kuremó, which is a species of Liana. The Wajãpi women use the stalk shavings of the Liana to make tea, which they use for headaches wetting their heads, and they use the macerate stalk shavings for body itching.

In Table 3, the signs and symptoms most reported by the women are described. Fever was the most commonly reported symptom. The full list is as follows: Fever (8 interviewees – 100% reported); Headache (7–87.5%); Abdominal ache (6 – 75%); Diarrhea (5–62.5%); Urination pain (3 – 37.5%); Curuba – body itching or scabies – (3–37.5%); Stomachache (2 - 25%); Post-partum bleeding – abnormal bleeding: (2 – 25%); Pain (contractions) during labor (3–37.5%); Malaria fever (3 – 37.5%); and snake bite (1 – 12.5%).

**Table 3:** The signs and symptoms most reported by Wajãpi Women.

**Illness**	**Total**	**Relative Frequency (%)**
Fever	8	100
Headache	7	87.5
Abdominal Pain	6	75
Diarrhea	5	62.5
Urination Pain	3	37.5
Curuba	3	37.5
Labor pains (childbirth)	3	37.5
Malaria fever	3	37.5
Stomachache	2	25
Post-partum bleeding	2	25
Cobra bite	1	12.5
Others	2	25

### Methods of preparation and application

Throughout the study, the use of four different preparations of distinct medicinal plants was verified. Fifteen methods of internal administration and 13 methods of external application were reported. The term “decoction” was not used, given that “tea” was the term used to define what is generally technically designated as a decoction.

In the daily life of these women, tea is the most often used preparation in the studied region. This method is used with approximately 29.6% of the useful species. The next most commonly used methods are baths and maceration (25.9%), followed closely by *in natura* use, and finally, juice (3.7%) (Table 4). The responses of women who used these practices reveal the following:

"“You cut a piece of the bark of the Kadjurá Liana, more or less the width of three fingers, place it in a small cloth with water and allow it to boil (tea). Wait for it to cool a little and drink it cold or hot. Use for diarrhea with blood”. M_2_"

"“For stomach pain and fainting, collect the epazote leaves in a little bit of water in a bowl. Next, drink it, and for fainting, smell it”. M _7_"

"“For urinary pain, cut the root of the Tapereba. Inside the root there is water; drink it directly (in natura).” M _5._"

**Table 4:** Uses of plant preparations as medicine by Wajãpi Women.

**Type of Preparation**	**Number of Preparations**	**Relative Frequency (%)**
Tea	08	29.6
Bath	07	25.9
*In natura*	04	14.8
Juice	01	3.7
Maceration	07	25.9
Total	27	100

Contact with indigenous women accentuated the perception that traditional medicine is rooted in oral transmission, without the interference of a medical institution, once these women have profound knowledge of herbs and medicinal plants. From the scientific point of view, this local knowledge is only validated when it is subjected to analysis and incorporated into an official system. However, for those women, the validation of these practices rests in what their ancestors passed on to them, and this learning has always achieved positive results when put into practice.

### Narratives: illness and treatment: has indigenous and non-indigenous knowledge been integrated?

The narratives of the interviewees on this topic were recorded and field observations were used to verify whether they report an efficacious cure or treatment of sickness and whether unique knowledge or integration occurred at the moment of need.

The theme of illness and cure can have diverse meanings within the same society, or in this case, between indigenous and non-indigenous societies. The knowledge has been accumulated and tested several times, and its practice has been updated.

In indigenous society, there are further unique characteristics. In addition to being accumulated in people’s memory, plant knowledge is accommodated in an integrated manner with adopted knowledge and is created and developed by the people. It is a cultural art form, and culture is known to be formed by practices, values and beliefs that are slowly developed over time.

For the Wajãpi women, the process of becoming ill has been culturally related to a cosmological and spiritual vision of non-human aggressors who do not respect rules or come into contact with secretions, objects, and people [2]. However, this has been changing slowly due to intervention of health professionals in the community.

In this way, traditional knowledge does not constitute an autonomous sector, but it is similar to the biomedical sector of Western societies with regard to diagnosing illnesses using their signs and symptoms. Becoming ill occurred when a life form, or species, became lodged in the body [13]. Treatment was carried out at home and was considered the best cure available because hospitals were considered at that time to be full of pestilence, and in hospitals, illness ran the risk of losing its normality.

Under this definition of illness, there was a movement from considering illness to be a specie regarding it as a symptom. In support of this change, symptoms came to be considered a pathological essence because it was clinically discovered that symptoms identify illnesses [14].

Despite the fact that conceptions regarding illness as a collection of symptoms were previously intertwined, there is still an apparent distance between methods of diagnosis and treatment, and the key to understanding them depends on the socio-cultural context.

It is important to emphasize that when speaking about illness, the women remain oriented towards the practice embodied in the teachings of their ancestors:

"[…] fever, diarrhea, and when we have a baby and we are recovering, we can not take a bath in the river; we menstruate, and then “mãe d”água” makes a lot of noise in our ears like a buzzer, as if it were summer; your head hurts a lot, lack of air, and it can cause you to faint”. (M_1_ )"

The cure for illness is connected to shamanism although treatment is first provided by family members (in this case, the Wajãpi women who provide care using medicinal plants). The shaman will intervene after being called by the family or whenever necessary, without invalidating the care these women provide.

This understanding of the ill person and his or her treatment in the Wajãpi culture is not understood by developed society, which complicates inter-ethnic relationships in various contexts, not only in health, but also in other related areas. In almost all cultures, most primary health assistance occurs within the family [15]. In the informal sector, women (mothers and grandmothers) are primarily responsible for medical assistance. These women always play a central role. In the Wayana-Aparai culture, women manipulate and cultivate medicinal plants around their homes as part of their therapeutic practice [16].

The situation above is similar to Wajãpi practices. However, in this study, when the female participants were asked, “When people fall ill in your community, to where do they go first for treatment?” The women responded,

"“Young Indians go to the Funasa pharmacy, they do not look for home remedies, they think it is easier and faster, they do not wait to do it”. (M_4_)"

"“Young Indians go straight to the pharmacy. As a child, the mother does it. Young people go to the pharmacy because it is easier and has a rapid effect. Many times the cure is far away, so it takes longer. When the medicine from the pharmacy does not treat it, we also try to treat it.”(M_1_)"

The acceptance of Western medicine is based on the power of industrialized drugs to treat illnesses, representing the dominance of the biomedical model that is integrated into every level of their societies. However, this vision does not represent a break with traditional behavior but, rather an adaptation of a society in contact with modern culture.

Among the Wajãpi, external illnesses are defined as those appearing after contact and named as “white disease” (karaiko) and should be treated as such, outside of the medical practices of the group. However, this does not mean that treatment only takes place through the use of chemicals, as M_2_ reports:

"“First they go to the FUNASA pharmacy because now they have medicine. When the medicine does not cure it, they look for the medicine that we make. When there is not a pharmacy, the “young Indians” come to us and use what we make. An illness caused by a python is only treated by the shaman. The shaman doesn’t treat malaria or diarrhea, but only illness from a python; if your head becomes dizzy, the shaman treats it”."

It is worth noting that Western medicine emerged from a vast reservoir of traditional and popular cures and subsequently spread to the rest of the world (thereby being transformed into the basic biomedical approach) [17]. Therefore, we examined whether these traditional cures were accepted by health professionals that worked in indigenous areas, even though the habits of Indians and health professionals are different.

Here it is evident that the Wajãpi women attempt to use medicinal plants to treat their illnesses. When this occurs, is there dialogue regarding these practices with professionals? Do the professionals discuss with the women how to use the medicines? Which plants are considered medicinal? The responses were listed below:

"“No, they don’t talk. I do not speak the Portuguese language well, and the health karaiko does not speak the Wajãpi language.” (M_7_)."

"“No, we do not talk. I do not know very much, and I believe that they do not know how the Indian lives” (M_1_)."

"“No, we do not talk. Why talk if the karaiko is often moved from the ambulatory?” (M_2_)"

Previous reports suggest that it can be understood that everyone has ways of life that are adapted to a particular place and culture, depending on their needs. However, in order to understand others, it is necessary to set aside our preconceptions and attempt to view the experiences of others in relation to their own understandings [18]. Through the responses of these women, it is clear that they are not curious about becoming familiar with the different methods of curing diseases. There was no dialogue regarding the language and communication barriers (cultural differences), and no bond was established because the professionals did not remain for long in these areas due to the lack of structures to facilitate their adaptation. The cultural barriers that were experienced in this case were not solely related to the cultural habits of the Indians; they are also related to the biomedical culture of the health professionals created by their training [19].

The indigenous women understand the importance of the health services of Western society; however, this interaction has to be viewed based on the experience of illness and cure of both groups, the indigenous women and the researchers. Appreciation for the treatment practices of indigenous women using medicinal plants leads to greater visibility of indigenous practices of healthcare. On one hand, this visibility seeks to create internal appreciation of these particular practices, especially among the younger population. On the other hand, the continuity of traditional knowledge seeks to encourage dialogue among different cultural groups.

### Traditional knowledge and sustainability

Among the many works describing the day-to-day life of indigenous women, we highlighted in this study the traditional knowledge and practices regarding the use of medicinal plants with Wajãpi.

Upon gaining knowledge about the important work of these women and their trajectory, it is necessary to remember that these women play a fundamental role in society, without which the domestic economy would not follow its normal course despite its many challenges. However, with regard to the economy, we are not referring to the accumulation of wealth but, rather to the richness of knowledge collected throughout the years.

This study examines traditional knowledge and how it can be sustained in an indigenous society. Therefore, the question of sustainability should be considered vital, not simply a public policy but an Indigenous Policy, sustaining their culture and providing better quality of life [20].

This study reports the relationships developed during the transmission of traditional knowledge needed for the maintenance of the social organization. During the time spent with these women, we noticed the knowledge received from their ancestors was naturally transmitted, based on the following responses received after we asked how knowledge regarding medicinal plants was acquired:

"“Through my father and my mother, I did not see my grandmother, and I wanted to learn. When my mother made medicine, I watched her or she called me. Each woman makes it for her family. When the mother is not there, we also make it for sick people in other families”. M_1_"

"“My father was the one who taught me; my mother died when I was a very young child.” M_6_"

The knowledge is obtained from older family members regardless of gender and is not restricted to a mere repertoire of medicinal herbs. Neither does it consist of a list of plant species. In reality, the knowledge includes formulas and understanding of the appropriate processes used to transform the plants, including their magic, ritual, and symbolic aspects.

One of the threats to the maintenance of traditional plant practices within indigenous communities is the possibility of not passing on this knowledge. Internal conflicts exist, mainly within the younger indigenous because their contact with developed society and question the knowledge of the ancients. Some even avoid wearing material markers of their traditions to avoid prejudice from non-Indian society, which is still present in daily life [19].

Upon questioning whether the women are passing their practical knowledge of medicinal plants to the younger population, the following responses were obtained:

"“I don’t pass it on, I don’t share it. When they call for me to teach others from their villages”, they say: “Is this really going to cure? Only medicine from the karaika cures”. “M_2_”"

"“I am not teaching anyone”. “M_5_”"

"“I pass it on to all of my daughters. For the others, I don’t know”. “M_3_”"

This evidence supports the importance of this research. It is clear that dedication to deepening these studies and to providing knowledge regarding how to evaluate the impact of long-term processes of change that can be “evaluated” by the members of the society themselves is needed. However, the Wajãpi do not share this view, and they remain at a standstill. The Wajãpi, concerned about the appreciation of the knowledge of the older population by adolescents, some leaders, and bilingual professors, look for alternatives and support reversing the current trend [21].

Given these facts, traditional knowledge contributes to indigenous subsistence and has been incorporated into the concept of sustainability. Indigenous communities possess an “environmental ethic” that not necessarily is universal to all indigenous communities. Values and specific characteristics are emphasized, such as family bonds and communication between generations, including ancestral connections, cooperation, concern for the well-being of future generations, local governance, self-sufficiency and dependence on natural local resources, opposition to the exploitation of resources, and respect for the natural environment, especially sacred sites [21].

## Conclusion

Indigenous women combine in their traditional knowledge a vast wisdom regarding medicinal plants that are used in the treatment and prevention of illnesses of the body and soul. For these women, the concept of illness and health is rooted in their cosmological vision, and their practices envision illness as the result of imbalance between beings and the environment in which they are situated. For them, well being for any living being means being healthy, whether or not illness is present. These women provide care for fever, headache, abdominal pain, diarrhea, urinary pain, and other illnesses. The plants that were most frequently identified for use in treatment belong to the following families: Leguminosae-Caesalpinoideae, Anacardiaceae, Meliaceae, and Rubiaceae. It is valid to note that since the Rain Forest has been awarded worldwide as a region with several medicinal species; few were detected in this study. The main reason for this is the use of industrialized medicines provided by the government and the interaction between both societies. Finally, if the use of medicinal plants decreases significantly by the societies which use it, the medical anthropology and ethnopharmacology will have a significant decrease in the data collection.

## Competing interests

The authors declare that they have no competing interests.

## Authors’ contributions

NDSM – Ethnobotanical and ethnopharmacological datas, analysis of taxonomic aspects; RSS - Literature survey and interpretation; FFP - Ethnobotanical and ethnopharmacological datas, writing of the manuscript, literature survey and interpretation and JCTC - Writing of the manuscript, literature survey and interpretation. All authors read and approved the final manuscript.
